# Effect of Screen Time on Social, Emotional, and Behavioural Problems in Children at a Tertiary Care Centre

**DOI:** 10.7759/cureus.116955

**Published:** 2026-09-26

**Authors:** Romana Wani, Pradeep Kumar, Bindu T Nair, Rajeev K Thapar, Praneta Swarup, Smita Nair

**Affiliations:** 1 Paediatrics, Sharda University School of Medical Sciences and Research, Greater Noida, IND; 2 Paediatrics and Child Health, Sharda University School of Medical Sciences and Research, Greater Noida, IND

**Keywords:** child behavior checklist (cbcl), externalizing problems, internalizing problems, preschool children, screen time

## Abstract

Background

Electronic screen-based media use among children has surged globally due to rapid technological advances, wide device availability, and evolving family routines. Excessive screen time affects cognitive development by reducing executive functioning, impairing memory retention, and limiting problem-solving. Behavioral problems, such as hyperactivity, aggression, and attention disorders, are also linked to excessive screen use.

Methods

A cross-sectional analytical study was conducted among 118 children aged 18 months to five years attending a tertiary care pediatric OPD over 18 months. Eligible children were enrolled by consecutive sampling. Screen exposure was assessed using the "Seven in Seven" questionnaire, categorized as high (≥7) or low (<7). Behavioral problems were evaluated using the Child Behavior Checklist for Ages 1.5-5 (CBCL/1.5-5), administered to all 118 children (confirmed against the master chart). Associations between screen exposure, behavioral outcomes, and risk factors were analyzed using the chi-square test or Fisher's exact test when the expected cell count was <5. A p-value of ≤0.05 was considered statistically significant. Standard multivariable logistic regression was unusable due to quasi-complete separation between screen exposure and several covariates, so Firth's penalized-likelihood logistic regression was used instead to identify independent factors, reporting adjusted odds ratios (aOR) and 95% CIs.

Results

Mean age was 3.59 ± 1.27 years; 67.8% were boys. Mean screen time score was 8.54 ± 2.99. CBCL classification is as follows: 62.71% clinical, 19.49% borderline, and 17.80% normal. The high exposure group (n = 78, 66.1%) is the following: 72 (92.3%) clinical, 5 (6.4%) borderline, and 1 (1.3%) normal; the low exposure group (n = 40, 33.9%) is the following: 20 (50.0%) normal, 18 (45.0%) borderline, and 2 (5.0%) clinical (χ² = 87.60, df = 2, p < 0.001). Externalizing problems (59.4%) predominated over internalizing (28.3%). On bivariate analysis, high exposure was significantly associated with lower physical activity (Welch t = 7.83, df = 60.7, p < 0.001), father's education (FET = 86.41, p < 0.001), mother's education (FET = 56.71, p < 0.001), father's occupation (FET = 110.85, p < 0.001), mother's occupation (FET = 69.11, p < 0.001), and family type (FET = 52.69, p < 0.001; all Fisher's exact given sparse cells); residence (p = 0.841) and sibling presence (p = 0.054) were not significant. In multivariable Firth regression, only father's occupation remained independent (skilled vs unskilled: aOR = 133.1, 95% CI: 4.3-4099.8, p = 0.005; professional vs unskilled: aOR = 577.1, 95% CI: 10.3-32198.4, p = 0.002); physical activity, father's education, family type, and sibling presence lost significance after adjustment.

Conclusions

In this cross-sectional, tertiary-care OPD-based sample, high screen exposure was significantly associated with clinically significant behavioral problems in preschool children, predominantly externalizing symptoms. Given the cross-sectional design and referral-based sampling, findings should be interpreted as associational rather than causal and are most generalizable to symptomatic children presenting to tertiary care rather than the general preschool population.

## Introduction

Over the past few years, children's exposure to screen-based electronic media has risen sharply worldwide, driven by faster technological change, greater access to digital devices, and shifting patterns of family life [1]. Screen time is generally understood as time spent using digital technologies - mobile phones, tablets, television, computers, laptops, and video games [1,2]. Children are now being exposed to screens at progressively earlier ages, effectively making them the first truly "digital native" generation. Evidence suggests that screen exposure among children has doubled in the last 20 years, with some infants beginning to interact with digital media as young as four months of age [3].

Both the Indian Academy of Pediatrics (IAP) and the American Academy of Pediatrics (AAP) advise no screen exposure at all for children below two years, and no more than one hour daily of supervised, high-quality educational content for those between two and five years [4]. Among children aged 6-12 years, screen use is expected to be carefully limited so that it does not compromise essential domains, such as sleep, physical activity, and interpersonal engagement [5]. However, in practice, a large proportion of Indian children continue to exceed these recommended limits, underscoring a persistent gap between guideline and behavior [6].

There is growing evidence that excessive screen exposure compromises cognitive development - weakening executive function, memory, and problem-solving skills [7]. Children who spend more than two hours a day in front of screens before age two have been found to score lower on cognitive assessments by age five [8]. Screen use also appears closely tied to social-emotional health: children with heavier screen exposure show more emotional dysregulation, social withdrawal, and anxiety [9]. Heavy consumption of fast-paced digital content, in particular, has been associated with elevated depression, loneliness, and diminished social skills, especially in older children [6].

Excess screen use has similarly been linked to behavioral difficulties, including hyperactivity, aggression, and attentional problems [10]. One key mechanism appears to be sleep disruption - screen use, especially near bedtime, delays sleep onset, fragments sleep architecture, and shortens overall sleep duration [11]. This shortened sleep, in turn, is tied to weaker self-regulation and a greater likelihood of behavioral problems [6]. The strength and consistency of these associations vary across populations, measurement tools, and the degree of adjustment for socioeconomic and family confounders, and not every study finds a significant effect once such factors are controlled for.

More recent work points to a bidirectional relationship between screen exposure and developmental or behavioral difficulties [12,13]. Children who already show cognitive, behavioral, or emotional challenges may be handed screens more often as a way to soothe them, which can deepen their reliance on digital media and worsen underlying symptoms [14]. Notably, children with hyperactivity, autism spectrum disorder, or attention difficulties tend to have greater screen exposure, as caregivers turn to screens as a behavior-management tool - a pattern some authors have proposed, though not conclusively demonstrated, may create a self-reinforcing cycle between screen use and dysregulation [10,15].

Although international literature has established links between heavy screen exposure and emotional dysregulation, attention deficit hyperactivity disorder (ADHD)-like symptoms, and broader neurodevelopmental outcomes, relatively few Indian tertiary care studies have combined a validated problematic-screen-exposure tool with a standardized behavioral instrument (Child Behavior Checklist (CBCL)) and a structured assessment of family-structure risk factors within the same preschool cohort, although related Indian work has examined screen exposure and behavioral outcomes using other tools [16-18]. Against this backdrop, the present study set out to examine the relationship between screen exposure (measured using the Seven-in-Seven Questionnaire [17]) and CBCL-based behavioral outcomes [18] in children between 18 months and five years of age, while identifying sociodemographic, family, and lifestyle factors that could guide counseling and preventive approaches in day-to-day pediatric care.

Thus, the primary objective of the study was to determine the association between screen exposure category (Seven-in-Seven score, high vs. low) and CBCL for Ages 1.5-5 (CBCL/1.5-5) behavioral classification (normal, borderline, clinical) in children aged 18 months to five years attending a tertiary care pediatric OPD. The secondary objectives were to identify the sociodemographic, family-structure, and lifestyle factors (including physical activity) associated with high screen exposure in this population, individually and after multivariable adjustment.

## Materials and methods

This observational cross-sectional study was conducted over 18 months in the Department of Pediatrics at the School of Medical Sciences & Research, Greater Noida, India. The study population comprised children aged 18 months to five years attending the pediatrics OPD at a tertiary care centre in North India. Children were enrolled from consecutive OPD attendees meeting eligibility criteria during the study period.

Ethical approval was obtained from the Institutional Ethics Committee prior to the commencement of the study (Ref. No. SU/SMS&R/76-A/2024/165). Written informed consent was obtained from parents/guardians of all enrolled children.

Methodology

The principal investigator collected sociodemographic and family information (age, sex, residence, parental education and occupation, family type, and sibling status) and lifestyle variables (physical activity) using a structured pediatric proforma. Physical activity was recorded as parent-reported average daily hours of active play/outdoor activity; like screen exposure, this measure was not independently validated, which is acknowledged as a limitation below. Parental occupation was classified descriptively as skilled, unskilled, or professional, and family type as nuclear (parents and children only), joint (parents and children co-residing with the families of siblings), three-generation (grandparents, parents, and children co-residing), or broken (single-parent household following death, divorce, or separation).

Screen exposure was assessed using the "Seven-in-Seven" Screen Exposure Questionnaire. The details of the Seven-in-Seven Questionnaire and its scoring are given in the Appendix (Supplementary Tables S1 and S8) [17]. This tool evaluates daily screen exposure, co-viewing with parents, limit setting, exposure during meals and before bedtime, age of initial screen exposure, and viewing of low-quality content. Scores range from 0 to 13 and were categorized as high (≥7) and low (<7) to identify potentially problematic exposure patterns.

The CBCL for Ages 1.5-5 (CBCL/1.5-5) was administered to all 118 enrolled children, irrespective of screen exposure category or presenting concern. The details of the CBCL cut-off ranges and item-domain mapping are given in the Appendix (Supplementary Tables S2-S3).

The CBCL, developed by Thomas M. Achenbach, is a DSM-oriented scale comprising 99 items that screen for internalizing problems (anxious/depressed, withdrawn/depressed, somatic complaints), externalizing problems (rule-breaking, aggression), and total problem scores. It also includes an "Other Problems" list for specific issues such as overeating and sleep disturbances. Permission was obtained for using this tool [18].

The CBCL was completed by parents/caregivers (and in some instances by clinicians), a mixed-rater approach that can introduce inter-rater measurement inconsistency and is acknowledged as a limitation below, and typically required 15-20 minutes. Children flagged by CBCL were further assessed by a clinical psychologist.

Sample size

The sample size was calculated based on the prevalence of excessive screen time reported by Varadarajan et al. (73%) [12]. The calculation used the formula:

*N = Z&sup2; &times; P(1&minus;P) / E&sup2;*

Here, Z represents the z-statistic for a 95% confidence level (1.96), P represents the expected prevalence (73%), and E represents the margin of error (8%). The calculated sample size was 118 children. This formula estimates a single proportion and was appropriate for establishing the expected prevalence of high screen exposure.

Statistical analysis

Collected data were entered into Microsoft Excel (Microsoft® Corp., Redmond, WA) and analyzed using Statistical Product and Service Solutions (SPSS, version 22; BM SPSS Statistics for Windows, Armonk, NY). Normality was evaluated using the Kolmogorov-Smirnov test. Continuous variables were summarized as mean ± standard deviation (SD) and median (interquartile range) as appropriate, while categorical variables were expressed as frequencies and proportions. Associations between categorical variables were tested using chi-square for contingency tables in which all expected cell counts were ≥5, and Fisher's exact test for any table containing an expected cell count <5. A p-value ≤0.05 was considered statistically significant. Standard maximum-likelihood binary logistic regression was not usable for the multivariable risk-factor analysis because screen exposure is quasi-completely separated from several covariates in this dataset (near-perfect prediction in some strata), which prevents standard logistic regression from converging to stable estimates. Firth's penalized-likelihood logistic regression, an established bias-reduction method, was used instead, with high screen exposure (≥7) as the dependent variable and physical activity, father's education, father's occupation, family type, and presence of siblings as covariates. Adjusted odds ratios (aOR) with Wald 95% confidence intervals are reported.

## Results

A total of 118 children were included: 80 (67.8%) males and 38 (32.2%) females. The median age was four years, with an age range of 1.5-5 years and an interquartile range (IQR) of 3.0 years. Mean age was 3.57 years (SD = 1.27), a minor correction from the originally reported 3.59. Median height was 101 cm (IQR: 19 cm), and mean height was 99.3 cm (SD = 11.39). Median weight was 14.4 kg (IQR: 6.18 kg), and mean weight was 14.64 kg (SD = 3.95). Median BMI was 14.5 kg/m² (IQR: 2.7), and mean BMI was 14.61 kg/m² (SD = 1.93).

Fathers most commonly had graduate/diploma education (33.90%) or postgraduate/PhD (31.36%), whereas 16.10% were uneducated. Among mothers, 27.11% had graduate/diploma education, 22.03% postgraduate/PhD, and 22.88% were uneducated. Regarding occupation, 44.07% of fathers were skilled, 32.20% unskilled, and 23.73% professionals; among mothers, 52.54% were skilled, 38.98% unskilled, and 8.47% professionals (occupational category definitions in Methods). Most children resided in urban areas (66.10%). Family type distribution showed 37.29% nuclear, 23.73% three-generation, 22.88% joint, and 16.10% broken families (definitions in Methods). The demographic characteristics and anthropometric profile of the study population are detailed in Table 1.

**Table 1:** Demographic and anthropometric profiles of the study population (n = 118)

Variables	n	%
Gender		
Male	80	67.8
Female	38	32.2
Anthropometry	Mean ± SD	Median (IQR)
Height (cm)	99.3 ± 11.39	101 (19)
Weight (kg)	14.64 ± 3.95	14.4 (6.18)
BMI (kg/m²)	14.61 ± 1.93	14.5 (2.7)
Education of the father		
Graduate/Diploma	40	33.9
PhD/Postgraduate	37	31.36
Primary/Secondary	22	18.65
Uneducated	19	16.1
Education of the mother		
Graduate/Diploma	32	27.11
PhD/Postgraduate	26	22.03
Primary/Secondary	33	28
Uneducated	27	22.88
Occupation of the father		
Skilled	52	44.07
Unskilled	38	32.2
Professional	28	23.73
Occupation of the mother		
Skilled	62	52.54
Unskilled	46	38.98
Professional	10	8.47
Residence		
Rural	40	33.9
Urban	78	66.1
Family type		
Broken	19	16.1
Joint	27	22.88
Nuclear	44	37.29
Three generation	28	23.73

The mean screen time score (Seven in Seven) in the overall sample was 8.54 (SD = 2.99). Children categorized as high screen exposure (≥7) had a mean score of 10.36 (SD = 1.79, n = 78), whereas those with low exposure (<7) had a mean score of 5.00 (SD = 0.99, n = 40). On CBCL classification, 74 children (62.71%) were in the clinical range, 23 (19.49%) borderline, and 21 (17.80%) normal. The association between screen exposure and CBCL classification was statistically significant (p < 0.001).

In the high screen exposure group (n = 78), 72 (92.3%) were in the clinical range, 5 (6.4%) borderline, and 1 (1.3%) normal. In the low screen exposure group (n = 40), 20 (50.0%) were normal, 18 (45.0%) borderline, and 2 (5.0%) clinical (Table 2). Pearson χ² = 87.60, df = 2, p < 0.001 (likelihood ratio χ2 = 100.61, df = 2, p < 0.001; linear-by-linear association χ2 = 80.40, df = 1, p < 0.001; no expected cell count was below 5, minimum expected count = 7.12).

**Table 2:** CBCL classification by screen exposure category (n = 118)

Parameters	Low (<7)	High (≥7)	Total
Normal	20	1	21
Borderline	18	5	23
Clinical	2	72	74
Total	40	78	118

Among the 74 children in the clinical range, the majority exhibited externalizing problems (44, 59.4%), followed by internalizing problems (21, 28.3%). A smaller proportion demonstrated sleep-related difficulties (4, 5.4%) and other behavioral or emotional issues (5, 6.7%).

Risk factor analysis for high screen exposure identified several significant correlates. Physical activity was significantly lower among children with high screen time (1.72 ± 0.84 hours/day, n = 78) compared with low screen time (3.33 ± 1.15 hours/day, n = 40; Levene's test for equality of variances p = 0.026, so the following is reported rather than the equal-variance t): Welch's t = 7.83, df = 60.7, p < 0.001 (Figure 1). High screen exposure was also associated with father's and mother's education, father's and mother's occupation, and family type (all p < 0.001 by Fisher's exact test; see Table 3). Residence was not associated with screen exposure (Fisher's exact p = 0.841), and the presence of siblings was not significantly associated (Fisher's exact p = 0.054, borderline). The details of the multivariable Firth logistic regression analysis are given in Table 4.

**Figure 1:**
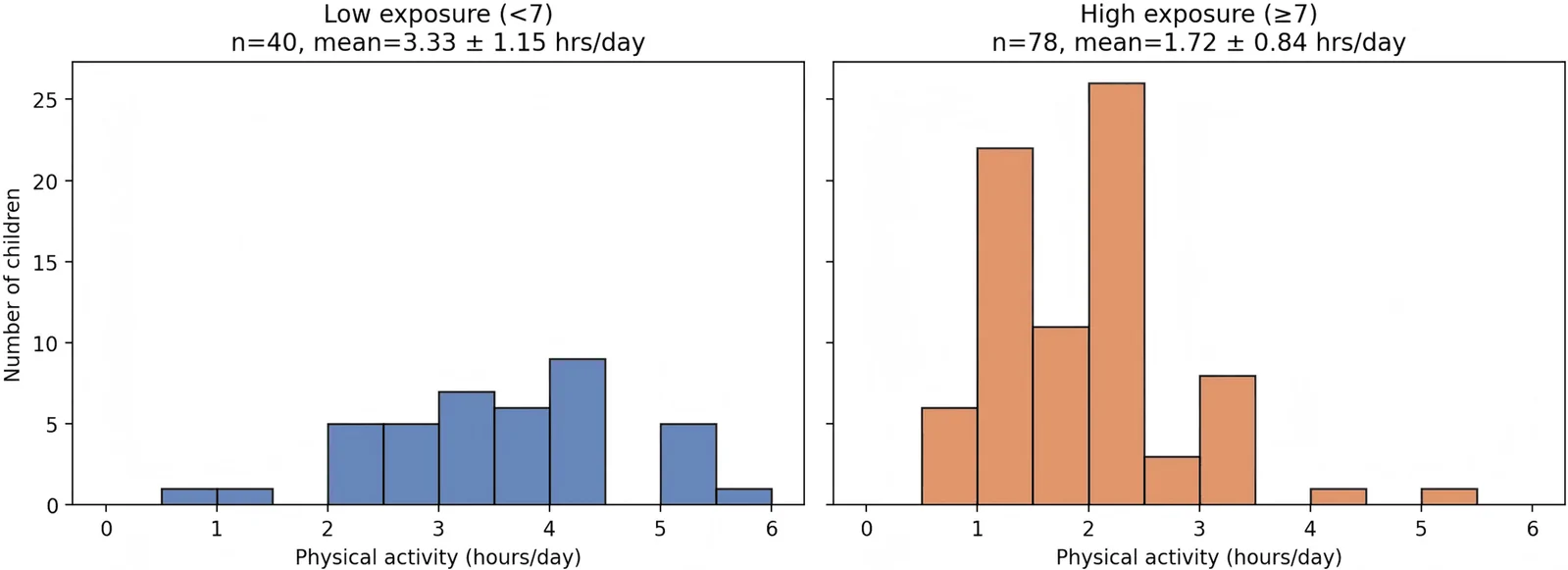
Distribution of daily physical activity by screen exposure category

**Table 3:** Risk factors associated with high screen time scores

Risk factor	Test used	Statistic	df	p-value
Physical activity (hrs/day)	Welch's t-test	t=7.83	60.7	<0.001
Father's education	Fisher's Exact Test	FET=86.41	3	<0.001
Mother's education	Fisher's Exact Test	FET=56.71	3	<0.001
Father's occupation	Fisher's Exact Test	FET=110.85	2	<0.001
Mother's occupation	Fisher's Exact Test	FET=69.11	2	<0.001
Residence	Fisher's Exact Test	χ²=0.05	1	0.841
Family type	Fisher's Exact Test	FET=52.69	3	<0.001
Presence of sibling	Fisher's Exact Test	χ²=3.87	1	0.054

**Table 4:** Multivariable Firth logistic regression for high screen exposure (≥7)

Variable	aOR	95% CI	p
Physical activity (per hour/day)	0.63	0.27-1.47	0.281
Father's education: Primary/Secondary (ref: Uneducated)	0.16	0.005-5.75	0.319
Father's education: Graduate/Diploma (ref: Uneducated)	1.6	0.06-45.5	0.783
Father's education: PhD/Postgraduate (ref: Uneducated)	0.16	0.003-8.28	0.358
Father's occupation: Skilled (ref: Unskilled)	133.1	4.3-4099.8	0.005
Father's occupation: Professional (ref: Unskilled)	577.1	10.3-32,198.4	0.002
Family type: Joint (ref: Nuclear)	1.25	0.07-22.5	0.882
Family type: Three-Generation (ref: Nuclear)	2.01	0.12-34.9	0.631
Family type: Broken (ref: Nuclear)	3.56	0.19-67.3	0.397
Presence of sibling: Yes (ref: No)	0.49	0.08-2.98	0.436

## Discussion

In this study, participants had a median age of four years, with boys forming the majority of the sample. A striking observation was that nearly two-thirds of the children fell within the CBCL clinical range, far higher than the roughly 10% clinical-range prevalence reported in general population-based samples using the same instrument. This gap is expected, not anomalous: recruitment took place through a tertiary-care pediatric OPD, which enriches the sample for children already presenting with developmental, behavioral, or parental concern. We attribute this elevated prevalence to referral-based sampling rather than to a selective-administration artifact. The prevalence figures in this study should therefore be read as describing a clinically referred population, not the general community prevalence of behavioral problems among preschoolers.

One of the central findings was a robust link between the category of screen exposure and behavioral classification. Children with heavy screen use were largely concentrated in the clinical range, while those with limited exposure fell mostly into the normal or borderline categories; father's occupation was the only sociodemographic factor independently associated with high exposure. This pattern lends support to the concern that extended or poorly monitored screen use in early childhood may be associated with difficulties in socio-emotional and behavioral regulation. Comparable links between screen exposure and behavioral difficulty have been documented in earlier work [10,19], although the wider literature on this topic is not unanimous, and studies vary in the strength of association reported after adjustment for socioeconomic confounders.

Among children in the clinical range, externalizing problems were more prevalent than internalizing ones. This indicates that behavioral dysregulation in preschoolers may more often present as outwardly directed symptoms - such as hyperactivity, impulsivity, irritability, or oppositional behavior - rather than as predominantly internalized emotional distress.

Several possible mechanisms could underlie the link between heavy screen use and behavioral difficulties. Greater screen time may plausibly crowd out active play, reduce meaningful caregiver-child interaction, and expose children to rapidly shifting visual stimuli that interfere with attentional control. Screen use close to bedtime may further disturb sleep patterns, shortening sleep duration and weakening behavioral regulation [6,11]. There is also evidence pointing to a bidirectional relationship between screen exposure and behavioral problems: children already showing behavioral difficulties may be given screens more often as a soothing strategy, which has been proposed to, in turn, increase exposure and potentially aggravate symptoms further [14,20]. Our cross-sectional design cannot distinguish between these directions of effect, or rule out shared underlying confounders.

Physical activity levels showed a marked inverse relationship with screen exposure. Children with greater screen use engaged in noticeably less daily physical activity than those with lower exposure. This reduction in physical activity may, in turn, limit chances for social engagement, outdoor play, and the development of self-regulatory abilities [3].

A notable and somewhat counterintuitive finding was the association between higher parental education and greater screen exposure. Although one might expect more educated parents to better adhere to recommended screen-time limits, this pattern could instead reflect greater access to digital devices and heavier reliance on digital media in households juggling demanding professional lives [17,21]. It is also possible that some parents view digital media as inherently educational, which may lead them to underestimate the cumulative impact of prolonged exposure. Parental education, occupation, and family type are correlated with one another in this sample; in a multivariable Firth logistic regression, only father's occupation remained independently associated with high screen exposure, indicating that the bivariate associations for education and family type reported above are better understood as reflecting their correlation with occupation than as independent effects.

Family structure emerged as another influencing factor. Children living in nuclear or broken families showed higher levels of screen exposure, while those from joint or three-generation households were more often represented in the low-exposure group. The presence of extended family members may offer additional supervision and create more opportunities for non-screen engagement [9,20]. Similarly, children without siblings showed higher exposure, suggesting that only children may have fewer chances for peer interaction within the home.

Place of residence did not show a meaningful association with screen exposure, suggesting that patterns of screen use may be fairly similar across urban and rural settings alike. This could reflect the growing reach of smartphones and digital media across geographic boundaries.

Strengths and limitations

This study carries several strengths, notably its focus on the preschool age group and its use of standardized tools to measure both screen exposure and behavioral outcomes [16,18,22]. That said, a considerably expanded set of limitations warrants mention too: (1) Recruitment through a tertiary-care OPD likely enriches the sample for symptomatic children, inflating clinical-range prevalence relative to the general population and limiting generalizability of the absolute prevalence figures. (2) CBCL was completed by a mix of parents/caregivers and, in some instances, clinicians, introducing potential inter-rater measurement inconsistency that was not formally assessed. (3) The blinding status of the clinical psychologist assessing flagged children to the child's screen-exposure category was not documented, introducing a potential risk of observer bias. (4) The sample-size formula used (single-proportion estimation) was not designed for the comparative analyses that form the primary aims of this study; a comparative, effect-size-based calculation would have been more appropriate. (5) Parental education, occupation, and family type are collinear; without multivariable adjustment, these should not be regarded as independent risk factors. Furthermore, the cross-sectional design of the study restricts any ability to draw causal conclusions about the relationship between screen exposure and behavioral outcomes.

## Conclusions

The present study demonstrated a significant cross-sectional association between high screen exposure and social, emotional, and behavioral problems among preschool children presenting to a tertiary care OPD. Most children with high screen exposure were classified in the CBCL clinical range, with externalizing problems as the most common presentation. On bivariate analysis, high screen exposure was associated with lower physical activity, father's and mother's education and occupation, and family type, but on multivariable Firth logistic regression, only father's occupation remained independently associated after adjustment, while residence and presence of siblings showed no significant association. These findings highlight excessive screen exposure as a correlate of, rather than a proven cause of, behavioral problems in this tertiary care population, and emphasize the importance of parental supervision, structured screen-time limits, and promotion of active play and interpersonal interaction in early childhood.

## Appendices

Supplementary Table S1

**Table 5:** Seven-in-Seven Screen Exposure Questionnaire - item scoring Total score range: 0-13. Classification: low <7, high ≥7 Source: Yalçin et al. [17], published under an Open Access Creative Commons Attribution 4.0 International License (CC BY 4.0), with no modifications to the questionnaire.

Item	Scoring
1. Daily screen time (weekday/weekend average, TV + other screens)	0 = <1 h/day; 1 = 1-2 h/day; 2 = >2 h/day
2. Viewing with parent(s) (co-viewing frequency)	0 = always; 1 = sometimes; 2 = rarely/never
3. Setting screen limits and child's compliance	0 = limits set and followed; 1 = no limits set; 2 = limits set but not followed
4. Screen use during meals	0 = no; 1 = yes
5. Screen use in the hour before bedtime	0 = no; 1 = yes
6. Age of first screen exposure	0 = ≥24 months; 1 = 18-23 months; 2 = 12-17 months; 3 = <12 months
7. Viewing of low-quality/inappropriate content	0 = age-appropriate/educational content only; 1 = one kind of inappropriate content; 2 = ≥2 kinds of inappropriate content

Supplementary Table S2

**Table 6:** CBCL/1.5-5 Interpretation guidelines and score ranges Reference table defining cut-off values for normal, borderline clinical, and clinical ranges across empirically based scales, including internalizing, externalizing, and total problem scores. CBCL/1.5-5: Child Behavior Checklist for Ages 1.5-5 Permission was obtained for using this tool [18].

Empirically Based Scale	Normal Range	Borderline Clinical Range	Clinical Range
1. Emotionally Reactive	0-5	6-8	9-18
2. Anxious/Depressed	0-6	7-8	9-16
3. Somatic Complaints	0-4	5-6	7-22
4. Withdrawn	0-4	5	6-16
5. Sleep Problems	0-7	8	9-14
6. Attention Problems	0-5	6	7-10
7. Aggressive Behaviour	0-20	21-24	25-38
Internalizing (Scales 1, 2, 3, 4)	0-13	14-17	18-72
Externalizing (Scales 6, 7)	0-20	21-24	25-48
Total Score	0-51	52-60	61-200

Supplementary Table S3

**Table 7:** CBCL/1.5-5 Empirically based scales - item mapping for scoring Tabulated classification of questionnaire items into empirically derived domains such as emotionally reactive, anxious/depressed, somatic complaints, withdrawn, sleep problems, attention problems, aggressive behavior, and other problems. CBCL/1.5-5: Child Behavior Checklist for Ages 1.5-5 Permission was obtained for using this tool [18].

Domain	Item Numbers
1. Emotionally Reactive	21, 46, 51, 79, 82, 83, 92, 97, 99
2. Anxious/Depressed	10, 33, 37, 43, 47, 68, 87, 90
3. Somatic Complaints	1, 7, 12, 19, 24, 39, 45, 52, 78, 86, 93
4. Withdrawn	2, 4, 23, 62, 67, 70, 71, 98
5. Sleep Problems	22, 38, 48, 64, 74, 84, 94
6. Attention Problems	5, 6, 56, 59, 95
7. Aggressive Behaviour	8, 15, 16, 18, 20, 27, 29, 35, 40, 42, 44, 53, 58, 66, 69, 81, 85, 88, 96
8. Other Problems	3, 9, 11, 13, 14, 17, 25, 26, 28, 30, 31, 32, 34, 36, 41, 49, 50, 54, 55, 57, 60, 61, 63, 65, 72, 73, 75, 76, 77, 80, 89, 91, 100

Supplementary Table S4

**Table 8:** Questions of the Seven-in-Seven Screen Exposure Questionnaire Source: Yalçin et al. [17], published under an Open Access Creative Commons Attribution 4.0 International License (CC BY 4.0), with no modifications to the questionnaire.

Sr. No.	Questions
1	How much time does your child spend: a) per a typical weekday looking at TV? b) per a typical weekday looking at other screen devices? c) per a typical weekend day looking at TV? d) per a typical weekend day looking at other screen devices?
2	What age did your child start using screen device?
3	Does your child use screen device during meals? □ Yes, he/she does □ No, he/she does not
4	Does your child use screen device at least for 1 h before bedtime? □ Yes, he/she does □ No, he/she does not
5	What are the types of programs and the kinds of screen content your child is exposed to?
6	Do you share screen media experiences with your child? □ Always □ Sometimes □ Rarely ever
7	Do you set screen limits? If you set them, does your child obey these limits? □ I set screen limits and my child obeys these limits. □ I do not set screen limits. □ I set screen limits but my child does not obey these limits.
